# Numeral Systems Across Languages Support Efficient Communication: From Approximate Numerosity to Recursion

**DOI:** 10.1162/opmi_a_00034

**Published:** 2020-08-01

**Authors:** Yang Xu, Emmy Liu, Terry Regier

**Affiliations:** Department of Computer Science, Cognitive Science Program, University of Toronto; Computer Science and Cognitive Science Programs, University of Toronto; Department of Linguistics, Cognitive Science Program, University of California, Berkeley

**Keywords:** number, semantic typology, efficient communication, functionalism, recursion

## Abstract

Languages differ qualitatively in their numeral systems. At one extreme, some languages have a small set of number terms, which denote approximate or inexact numerosities; at the other extreme, many languages have forms for exact numerosities over a very large range, through a recursively defined counting system. Why do numeral systems vary as they do? Here, we use computational analyses to explore the numeral systems of 30 languages that span this spectrum. We find that these numeral systems all reflect a functional need for efficient communication, mirroring existing arguments in other semantic domains such as color, kinship, and space. Our findings suggest that cross-language variation in numeral systems may be understood in terms of a shared functional need to communicate precisely while using minimal cognitive resources.

## NUMERAL SYSTEMS

A central question in cognitive science is why languages partition human experience into categories in the ways they do (Berlin & Kay, 1969; Levinson & Meira, 2003). Here, we explore this question in the domain of number.

Number is a core element of human knowledge (e.g., Spelke & Kinzler, 2007) and languages vary widely in their numeral systems (Beller & Bender, 2008; Bender & Beller, 2014; Comrie, 2013; Greenberg, 1978; Hammarström, 2010). Moreover, there are qualitatively distinct classes of such numeral systems. Some languages have numeral systems that express only approximate or inexact numerosity; other languages have systems that express exact numerosity but only over a restricted range of relatively small numbers; while yet other languages have fully recursive counting systems that express exact numerosity over a very large range. These different numeral systems are likely to be grounded in different cognitive capacities for judging numerosity. For example, approximate numeral systems may be grounded directly in the nonlinguistic approximate number system, a cognitive capacity for approximate numerosity that humans share with nonhuman animals (Dehaene, 2011). At the other extreme, the ability to judge exact high numerosity is not universal but appears instead to rely on the existence of a linguistic counting system that singles out such exact high numerosities (Gordon, 2004; Pica, Lemer, Izard, & Dehaene, 2004).

We seek to understand why certain numeral systems are attested in the world’s languages while other logically possible systems are not. We also seek to understand why the qualitative classes of such systems—from approximate counting, to exact counting over a restricted range, to fully recursive counting—appear as they do.

## EFFICIENT COMMUNICATION

An existing proposal has the potential to answer these questions. It has long been argued (e.g., von der Gabelentz, 1901; Zipf, 1949) that languages are shaped by functional pressure for efficient communication—that is, pressure to communicate precisely yet with minimal cognitive effort—and this idea has attracted increasing attention recently (e.g., Fedzechkina, Jaeger, & Newport, 2012; Gibson et al., 2019; Haspelmath, 1999; Hopper & Traugott, 2003; Kanwal, Smith, Culbertson, & Kirby, 2017; Piantadosi, Tily, & Gibson, 2011; Smith, Tamariz, & Kirby, 2013). Of direct relevance to numeral systems, it has been argued in particular that systems of word meanings across languages reflect such a need for efficient communication (Kemp, Xu, & Regier, 2018). On this account, for any given semantic domain, the different categorical partitionings of that domain observed in the world’s languages represent different means to the same functional end of efficiency. This idea has been supported by cross-language computational analyses in the domains of color (Regier, Kemp, & Kay, 2015; Zaslavsky, Kemp, Regier, & Tishby, 2018), kinship (Kemp & Regier, 2012), spatial relations (Khetarpal, Neveu, Majid, Michael, & Regier, 2013), names for containers (Xu, Regier, & Malt, 2016; Zaslavsky, Regier, Tishby, & Kemp, 2019), and names for seasons (Kemp, Gaby, & Regier, 2019). We ask whether the same idea explains why numeral systems appear as they do, from approximate to fully recursive form.

The idea of efficient communication involves a tradeoff between two competing forces: informativeness and simplicity. An informative system is one that supports precise communication; a simple system is one with a compact cognitive representation. A maximally informative system would have a separate word for each object in a given semantic domain—which would be complex, not simple. In contrast, a maximally simple system would have just one word for all objects in a given semantic domain—which would not support precise communication. The proposal is that attested semantic systems are those that achieve a near-optimal tradeoff between these two competing forces, and thus achieve communicative efficiency.

**Figure 1.**
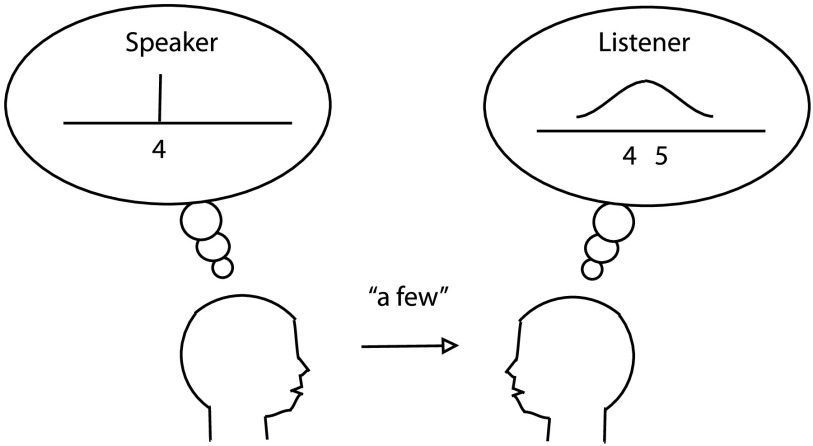
Scenario for communicating a number.

Figure 1 illustrates these ideas. Here, a speaker has a particular number in mind (4, mentally represented as an exact point on a number line), and wishes to convey that number to a listener. The speaker has expressed that number using the English approximate term “a few,” rather than the exact term “four” that is also available in English. On the basis of this utterance, the listener *mentally reconstructs* the number that the listener believes the speaker intended. Because the term “a few” is inexact, the listener’s reconstruction of the intended number is also inexact, and is shown as a probability distribution centered near 4 or 5 and extending to neighboring numbers as well. As a result, the listener’s mental reconstruction does not match the speaker’s intention perfectly. However, if the speaker had instead used the exact term “four,” that term would have allowed the listener to reconstruct the speaker’s intended meaning perfectly. We take the informativeness of communication to be the extent to which the listener’s mental reconstruction matches the speaker’s representation. Communication is not perfectly informative in the illustrated case of “a few” but would be perfectly informative in the case of “four.”

Clearly, an exact numeral system that picks out specific integers is more informative than an approximate system—but it is less simple. A system of approximate numerals can span a given range of the number line using very few terms, whereas many exact integer terms would be needed to span the same range. Thus, the high informativeness of an exact numeral system comes at a high cognitive cost. Importantly, however, a *recursive* exact system would be specifiable using a relatively small number of generative rules, rather than separate lexical entries for each exact numeral. Thus, recursive numeral systems may be a cognitive tool (Frank, Everett, Fedorenko, & Gibson, 2008) that enables highly informative communication about number at the price of only modest cognitive complexity (e.g., Piantadosi, Tenenbaum, & Goodman, 2012).

We wish to know whether these ideas can account for which numeral systems, and which classes of such systems, are attested across languages. To this end, we require: (1) a cross-language data set of numeral systems that captures the distinctions between classes of such systems, (2) a formal specification of our theory, and (3) a test of the theory against the data. We specify each of these in turn below, and then present our results. To preview our results, we find that numeral systems across languages tend to support near-optimally efficient communication, and that the drive for efficient communication also helps to explain why the different classes of numeral systems appear as they do. Our results suggest that the different types of numeral system found across languages all support the same functional goal of efficient communication, in different ways.

## CROSS-LINGUISTIC DATA

We considered the numeral systems of 30 languages, listed in Table 1, which span the spectrum from approximate to exact restricted to recursive numeral systems. We have used these class designations somewhat loosely up until now, and define them more precisely in the Supplemental Materials. The majority of the languages in this data set were drawn from Comrie’s chapter on numeral bases in the *World Atlas of Language Structures* (WALS) (Comrie, 2013). That chapter includes references to grammars for individual languages, each of which describes that language’s numeral system. We also considered the numeral systems of French and Spanish, and three languages (Chiquitano, Fuyuge, Krenák) from Hammarström’s (2010) survey of rare numeral systems. These numeral systems were supplemented by a description of the Mundurukú numeral system (Pica et al., 2004).

**Table 1.** The 30 languages in the data set, grouped by type of numeral system.

**Approximate systems (6 languages):**
Chiquitano, Fuyuge, Gooniyandi, Mundurukú, Pirahã, Wari’
**Exact restricted systems (18 languages):**
Achagua, Araona, Awa Pit, Barasano, Baré, Hixkaryana, Hup, Imonda, Kayardild, Krenák, Mangarrayi, Martuthunira, Pitjantjatjara, Rama, Waskia, Wichí, Yidiny, !Xóõ
**Recursive systems (6 languages):**
English, Mandarin, and Spanish (base 10), Ainu (base 20), French and Georgian (base 10 and 20)

## FORMAL PRESENTATION OF THEORY

We have seen that the notion of efficient communication involves a tradeoff between the competing forces of simplicity and informativeness. We first formalize each of these two forces in turn, and then the tradeoff between them (Kemp & Regier, 2012; Regier et al., 2015). Throughout this article, we restrict our attention to numerals over the range 1–100.

### Simplicity

Simplicity is the opposite of complexity, and we define the *complexity* of a numeral system to be the number of symbols needed to specify it. This notion of complexity is grounded in standard ideas from algorithmic information theory (e.g., Kolmogorov, 1963; Li & Vitányi, 2013). We specify numeral systems as grammars, expressed in a language of thought (Fodor, 1975; Piantadosi et al., 2012), and based on the primitive components listed in Table 2 and explained in the Supplemental Materials. The complexity of each system is thus given by the number of symbols in the corresponding grammar.

**Table 2.** Grammatical components for representing numeral systems.

**Component**	**Description**
c	Primitive concept c = 1, 2 or 3
$\tilde{x}$	Gaussian with approximate mean $\tilde{x}$
m (*w*)	Meaning of form *w*
s (*w*, *v*)	Successor of *w* with interval *v*; s (*w*) = s (*w*, 1)
h (*w*)	Higher than *w*
+	Addition
−	Subtraction
×	Multiplication
÷	Division
p (*x*, *n*)	*x* to the *n*th power
$\overset{d}{=}$	Form definition
∈	Set definition
≡	Equivalence

Tables 3, 4, and 5 present grammars for the numeral systems of three languages, one from each of the three classes we consider here, and indicate the complexity of each grammar. Different authors sometimes hold different views on the grammar of a given numeral system, and here we chose to work with grammars from representative sources, while acknowledging that such disagreement exists.^1^

**Table 3.** Grammar for Piraha (approximate) numeral system for the range 1–100.

**Number**	**Rule**	**Complexity**
1	‘hoi_1_’ $\overset{d}{=}$ $\tilde{1}$	3
∼2–4	‘hoi_2_’ $\overset{d}{=}$ $\tilde{3}$	3
∼5–100	‘aibaagi’ $\overset{d}{=}$ $\tilde{52}$	3
		Σ = 9

*Note*. Each rule is composed of symbols, and each symbol adds a unit complexity of 1. We do not use subitizing because work by Gordon (2004) suggests that the numeral for 1 is inexact in Piraha.

**Table 4.** Grammar for Kayardild (exact restricted) numeral system for the range 1–100.

**Number**	**Rule**	**Complexity**
1	‘warngiida’ $\overset{d}{=}$ 1	3
2	‘kiyarrngka’ $\overset{d}{=}$ 2	3
3	‘burldamurra’ $\overset{d}{=}$ 3	3
4	‘mirndinda’ $\overset{d}{=}$ s (‘burldamurra’)	4
5–100	‘muthaa’ $\overset{d}{=}$ h (‘mirndinda’)	4
		Σ = 17

*Note*. Each rule is composed of symbols, and each symbol adds a unit complexity of 1.

**Table 5.** Grammar for English (recursive) numeral system for the range 1–100.

**Number**	**Rule**	**Complexity**
1	‘one’ $\overset{d}{=}$ 1	3
2	‘two’ $\overset{d}{=}$ 2	3
3	‘three’ $\overset{d}{=}$ 3	3
4	‘four’ $\overset{d}{=}$ s (‘three’)	4
5	‘five’ $\overset{d}{=}$ s (‘four’)	4
6	‘six’ $\overset{d}{=}$ s (‘five’)	4
7	‘seven’ $\overset{d}{=}$ s (‘six’)	4
8	‘eight’ $\overset{d}{=}$ s (‘seven’)	4
9	‘nine’ $\overset{d}{=}$ s (‘eight’)	4
10	‘ten’ $\overset{d}{=}$ s(‘nine’)	4
11	‘eleven’ $\overset{d}{=}$ s (‘ten’)	4
12	‘twelve’ $\overset{d}{=}$ s (‘eleven’)	4
13…19	u‘teen’ $\overset{d}{=}$ m (u) + m (‘ten’)	8
20…90	u‘ty’ $\overset{d}{=}$ m (u) × m (‘ten’)	8
21…99	u‘ty’-v $\overset{d}{=}$ m (u) × m (‘ten’) + m (v)	13
100	‘hundred’ $\overset{d}{=}$ p (m (‘ten’), m (‘two’))	7
	u ∈ {‘twen’, ‘thir’, …, ‘eigh’, ‘nine’}	10
	v ∈ {‘one’, ‘two’, …, ‘eight’, ‘nine’}	11
	‘twen’ ≡ ‘two’	3
	‘thir’ ≡ ‘three’	3
	‘for’ ≡ ‘four’	3
	‘fif’ ≡ ‘five’	3
	‘eigh’ ≡ ‘eight’	3
		Σ = 117

*Note*. Each rule is composed of symbols, and each symbol adds a unit complexity of 1.

### Informativeness

Informativeness of communication was illustrated in the communicative scenario of Figure 1. Returning to that scenario, we may represent the speaker’s intended meaning as a probability distribution *S*(*i*) over numbers *i*, and analogously represent the listener’s mental reconstruction of that meaning as a distribution *L*_*w*_(*i*) over numbers *i*, based on the word *w* uttered by the speaker. We assume that the speaker is certain of the target number: *S*(*t*) = 1 for the intended target number *t*, and *S*(*i*) = 0 for all other numbers *i* ≠ *t* (Regier et al., 2015; cf. Zaslavsky et al., 2018). We assume that the listener distribution *L*_*w*_(*i*) depends on the number word *w* produced by the speaker, which may be grounded in primitives drawn from the subitizing number system, the approximate number system, or exact numerosity, as specified in the Supplemental Materials. We do not model pragmatic reasoning in which the listener and speaker reason recursively about each other (Brooks, Audet, & Barner, 2013; Frank & Goodman, 2012).

Given specifications of the speaker (*S*) and listener (*L*_*w*_) distributions, we define the communicative cost *C*(*t*) of communicating a target number *t* under a given numeral system to be the *information lost* in communication—that is, the information lost in the listener’s reconstruction *L*_*w*_ when compared to the speaker’s distribution *S*. We model this information loss as the Kullback-Leibler (KL) divergence between distributions *S* and *L*_*w*_. In the case of speaker certainty (*S*(*t*) = 1 for the target number *t*), this reduces to surprisal:^2^

$$C(t)=D_{KL}(S||L_{w})=\underset{i}{\sum }S(i)\log_{2}\frac{S(i)}{L_{w}(i)}=\log_{2}\frac{1}{L_{w}(t)}$$ (1)

We model the communicative cost for a numeral system as a whole as the expected value of *C* over all possible target numbers *t*:

$$E[C]=\underset{t}{\sum }N(t)C(t)$$ (2)

Here, *N*(*t*) is the need probability of target number *t*—that is, the probability that a speaker will need to refer to *t* rather than some other number. We estimated need probabilities by the normalized frequencies of English numerals in the Google ngram corpus (Michel et al., 2011) for the year 2000, as described in the Supplemental Materials. Qualitatively, this yields need that drops off with increasing numerosity. The distribution of need probabilities may well vary across languages and cultures, and would ideally be estimated on a per-language basis. However, we do not have data that would support such per-language estimation of need probabilities, and so we tentatively assume a universal distribution estimated from English usage (Kemp & Regier, 2012). The qualitative nature of this distribution—a dropoff in need as numbers increase—may generalize across cultures even if the specific quantitative shape of that dropoff does not (Dehaene & Mehler, 1992; Piantadosi, 2016). In our analyses below, we compare this corpus-based distribution with other hypothetical need distributions.

### Tradeoff

We take a numeral system to be simple to the extent that it exhibits low complexity, and we take it to be informative to the extent that it exhibits low communicative cost *E*[*C*]. Given this, we consider a numeral system to be *near-optimally efficient* if it is more informative (i.e., exhibits lower communicative cost) than most logically possible hypothetical systems of the same complexity, or if it is simpler (i.e., exhibits lower complexity) than most logically possible hypothetical systems that have the same communicative cost.

## STUDIES

We test our theory against the data in two steps. We first assess the semantic primitives in Table 2. We do so by testing whether the primitives that represent subitizing and the approximate number system can accommodate fine-grained linguistic data from the one relevant language for which we have such data, Mundurukú. We then use the full set of primitives to conduct efficiency analyses on all languages in our data set.

### Mundurukú and the Approximate Number System

**Figure 2.**
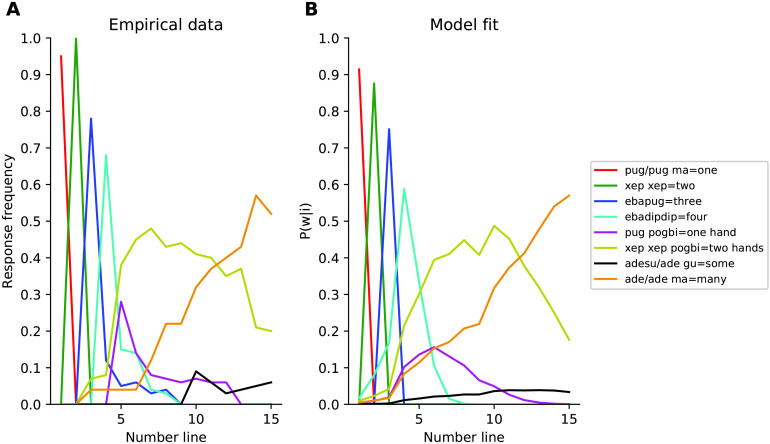
**Modeling Mundurukú naming data.** (A) Empirical data collected by Pica et al. (2004). (B) Fit to the empirical data of a model based on primitives that capture subitizing and Weber’s law.

Pica et al. (2004) showed that their formalization of the approximate number system, governed by Weber’s law, accounted well for *nonlinguistic* numerosity judgments by speakers of Mundurukú. They also collected fine-grained data on the way speakers of Mundurukú *name* different numerosities, but they did not directly test whether their formalization of the approximate number system also accommodates those linguistic data. We test that question here. Figure 2A shows empirical Mundurukú number naming data from Pica et al. (2004)—specifically, for numerosities 1 to 15, this figure shows the fraction of times each numerosity *i* was named with a given Mundurukú word or locution *w*. Figure 2B shows the fit to these data of a model based on subitizing and the approximate number system, grounded in the relevant semantic primitives from Table 2. The model fit was good (MSE = 0.002), and was superior to that of other models considered. Model details along with variants of the model using different Weber fraction values are provided in the Supplemental Materials. These findings suggest that the model of subitizing and the approximate number system given by the relevant semantic primitives in Table 2 provide a reasonable basis for grounding approximate numeral systems.

### Efficiency of Numeral Systems

We wished to test (a) whether all numeral systems in our data set are near-optimally efficient, and (b) whether the notion of efficiency also helps to explain the distinct classes of system that appear in the data.

**Figure 3.**
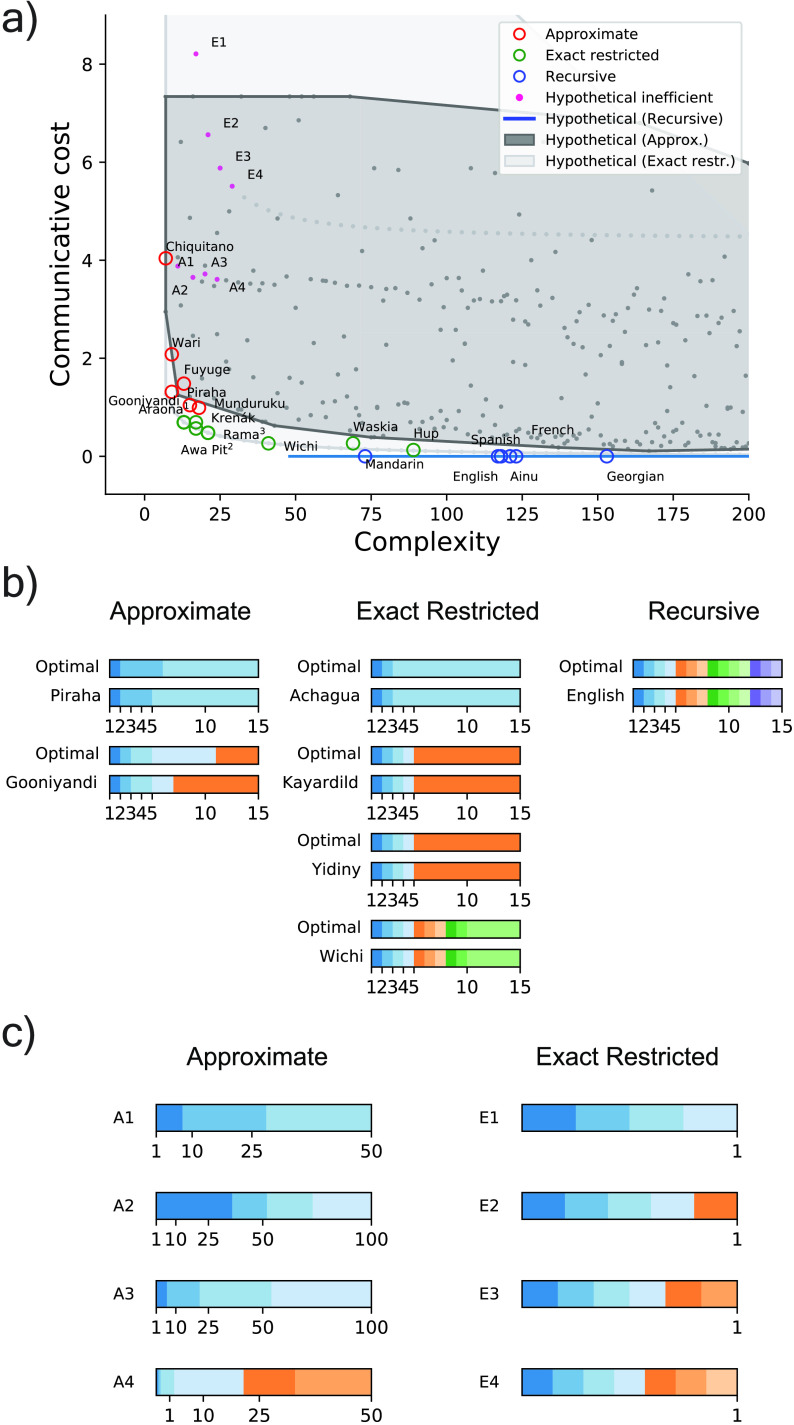
**Efficiency analysis of numeral systems.** (A) Near-optimal tradeoff between communicative cost and complexity across attested numeral systems, compared with corresponding hypothetical approximate, exact restricted, and recursive systems. Several exact restricted systems are equivalent here, namely, languages with terms for the first three numerals and a higher term (Araona, Achagua, Baré, Hixkaryana, Martuthunira, Mangarrayi, Pitjantjatjara, !Xóõ), terms for the first four numerals and a higher term (Awa Pit, Kayardild), and terms for the first six numerals and a higher term (Rama, Barasano, Imonda, Yidiny). (B) Comparison of sample attested systems to theoretically optimal systems of the same complexity. (C) Sample of nonoptimal hypothetical approximate (A1–A4) and exact restricted (E1–E4) systems, highlighted in pink in A. In B and C, each hue specifies the range of a corresponding numeral.

To test whether the attested numeral systems in our data set are near-optimally efficient, we assessed their simplicity and informativeness relative to a large set of logically possible hypothetical systems. These hypothetical systems fell in the same three major classes as our attested systems: approximate, exact restricted, and recursive. Details of these hypothetical systems are provided in the Supplemental Materials. Figure 3A shows sampled hypothetical systems (in dots), along with the convex hull of those sampled hypothetical systems, for approximate systems and exact restrictive systems, and the full set of hypothetical recursive systems, plotted according to their complexity and communicative cost, and compared with attested systems (shown as colored circles). The dark gray region denotes the range of costs exhibited by approximate hypothetical systems of various complexities; the light gray region denotes the range of costs exhibited by exact restricted hypothetical systems of various complexities; and the extent of the black horizontal line at communicative cost 0 denotes the range of complexities exhibited by hypothetical recursive systems, all of which have communicative cost 0. It can be seen that, in general, attested numeral systems in our data set tend to be more informative (show lower communicative cost) than most hypothetical alternatives of the same complexity. Thus, despite their variation, these attested systems all seem to share the capacity to support near-optimally efficient communication about number, suggesting that they may reflect adaptation for that function. In the Supplemental Materials, we show that these results are similar under alternative values of the Weber fraction.

Among the hypothetical recursive systems we considered, canonical base-10 (decimal) is one of the simpler systems. For example, Mandarin Chinese is a canonical base-10 system. The simplicity of base 10 reflects frequency of occurrence among the world’s languages (e.g., Comrie, 2013). In comparison, English as a variant of base-10 system (e.g., “eleven” and “twelve” have separate forms and do not derive their meanings from the base “ten”), and recursive systems with base 20 (e.g., Ainu) or a hybrid of bases 10 and 20 (e.g., Georgian) tend to be more complex. These findings are consistent with the suggestion (Ansuini, 2009; Hurford, 1987) that the relative complexity of various types of recursive system may partly explain the relative frequency of the appearance of such systems. We provide further detail on the relative complexities of canonical recursive systems in the the Supplemental Materials.

Figure 3B shows sample systems from our data set compared with the theoretically optimally informative (lowest cost) systems of the same complexity—in all cases color-coded such that a numeral corresponds to a colored region of the number line. It can be seen that the attested systems resemble these theoretical optima, again suggesting that the attested systems may have adapted to functional pressure to support efficient communication about number.

In contrast, Figure 3C shows example hypothetical numeral systems (for the range 1–100) that are further away from the optimal and attested numeral systems, with their exact positions indicated in Figure 3A. Although these systems are logically possible, they do not appear in real numeral systems and are generally inefficient because their extensions for the low-order numerals (e.g., those below 10) tend to be coarse. As such, these systems cannot disambiguate numerals that have the highest communicative need probabilities and therefore are highly uninformative.

**Figure 4.**
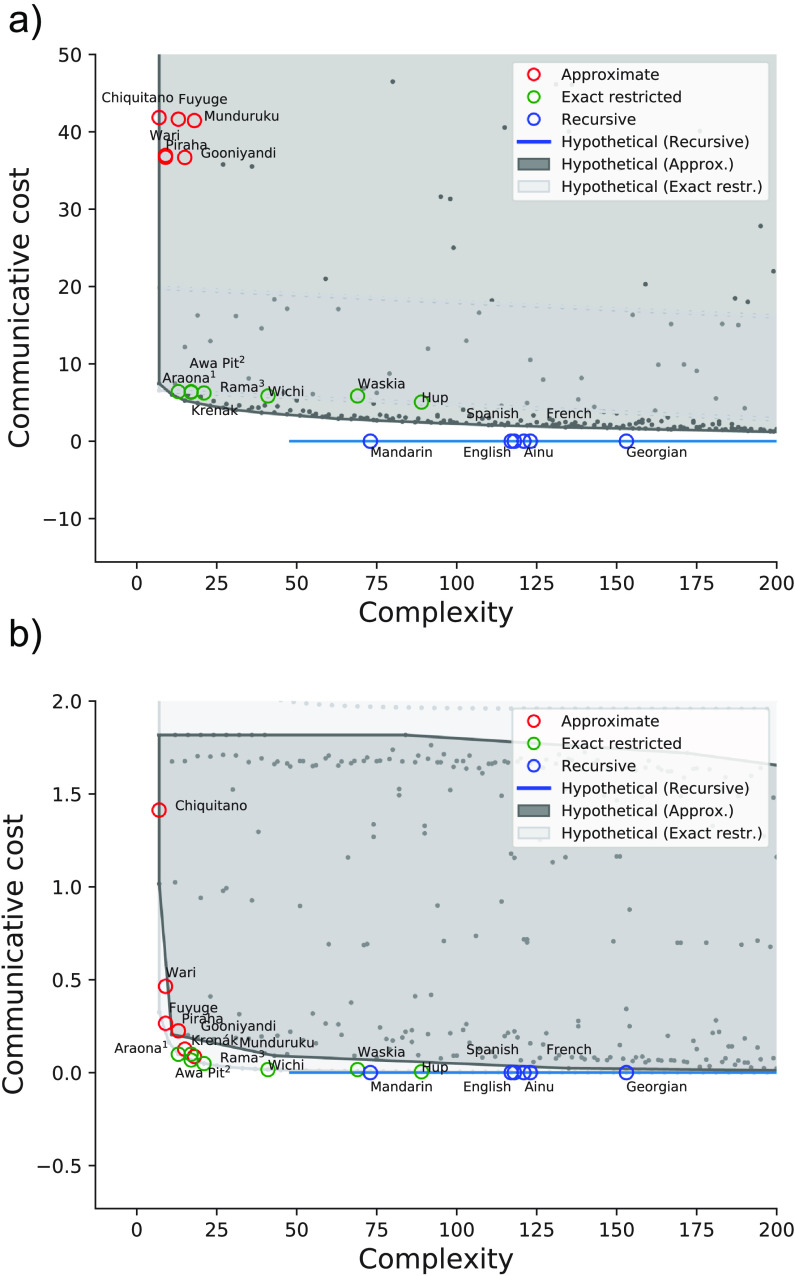
Efficiency of attested numeral systems in comparison to theoretical systems based on a uniform (A) and a left-skewed (B) need distribution.

To further examine how need probability influences the efficiency of numeral systems, we varied the need probability between extremes to assess its impact on the efficiency results. One extreme was a uniform distribution, as this would remove the advantage of placing exact terms at the beginning of the number line, increasing the cost for approximate and exact restricted systems. This can be seen in Figure 4A. Another extreme used was a distribution that was more left-skewed than the one based on corpus counts. This can be seen in Figure 4B. Using the uniform need probability, all hypothetical systems had higher communicative cost, and attested systems were further from the frontier as expected. Using the more skewed need probability, hypothetical systems were lower in communicative cost and attested systems were near-optimal as in the original case. This indicates that the efficiency of attested systems relies on the tendency for smaller numeric values to be used more often.

These findings suggest that the pattern of near-optimal efficiency is critically dependent on communicative need (Gibson et al., 2017; Kemp & Regier, 2012; Zaslavsky, Kemp, Tishby, & Regier, 2019a, 2019b). We obtain this pattern of near-optimality when assuming a need distribution that is based on corpus counts, and when assuming a steeper curve as might be expected for societies with less need to refer often to high numerosities. But we do not obtain this pattern of near-optimality when instead assuming a uniform need distribution, which is logically possible but seems intuitively unlikely to characterize the numeral need distribution for any society.

Our results also support a functional account of why the different classes of numeral system in the world’s languages appear as they do, namely, as qualitatively different ways of navigating the tradeoff between simplicity and informativeness. Approximate numeral systems (shown as red circles in Figure 3A) represent one extreme on a continuum: they are simple (noncomplex), requiring only a minimal cognitive investment in communicating about number. These systems support near-optimally informative communication for that level of cognitive investment—but they do not closely approach perfectly informative (0 cost) communication. Mundurukú is essentially poised at a tipping point between such approximate systems and exact restricted systems: it is the most complex and most informative of the approximate systems in our data. Exact restricted systems (shown as green circles in the figure) tend to be slightly more complex and support somewhat more informative communication. Finally, recursive systems represent the informative extreme of this continuum: these systems support perfectly informative communication, because there is a (recursively generated) separate name for each integer within a large range. Such fine-grained naming would be prohibitively expensive under a nonrecursive system: there would have to be one rule per integer in the range covered. But a recursive system supports perfectly informative communication over a large range, at the cost of only modest complexity.

## DISCUSSION AND CONCLUSION

We have seen that the need for efficient communication helps to explain why numeral systems across languages take the forms they do, by analogy with recent demonstrations in other semantic domains—and that the same functional need helps to explain the qualitatively different classes of numeral system found across languages. At the core of this explanation is the idea that attested numeral systems near-optimally trade off the competing demands of informativeness and simplicity, given a set of motivated semantic primitives, and a need distribution grounded in linguistic usage.

The semantic primitives on which we draw support both exact and approximate enumeration. An interesting connection in this regard, for which we thank an anonymous reviewer, is that the Weber-Fechner law, which characterizes the approximate number system, has itself been argued to reflect a process of informational optimization (Portugal & Svaiter, 2011; Sun, Wang, Goyal, & Varshney, 2012). This suggests that there may be related processes operating at different levels of a single numerical system.

Our results suggest that need probability plays a critical role in explaining why some logically possible partitions of the number line are not attested in the world’s numeral systems. In particular, our results suggest that the dominant need to refer to small rather than high numbers may explain why some numeral systems make fine distinctions among small numbers while supporting only imprecise enumeration for higher numbers. This coheres with the centrality of need probability in accounting for cross-language variation in other semantic domains, such as kinship (Kemp & Regier, 2012) and color (Gibson et al., 2017; Zaslavsky, Kemp, et al., 2019a, 2019b).

We have made a number of simplifying assumptions in our analyses, and future work can usefully explore alternatives to some of these assumptions. For example, our efficiency analyses have focused on one basic function of numeral systems, namely, the communication of number—but numeral systems also serve other important functions such as arithmetic calculation (Bender & Beller, 2014). Similarly, we have not explored the influence of physical tally systems, including those grounded in the human body, such as finger counting (Bender & Beller, 2012). Finally, we have assumed that cognitive complexity is well-captured by space rather than time complexity: we have focused on the representational cost of specifying a numeral grammar, rather than, for example, the amount of time it would take to derive numeral forms using such a grammar. Whether and how our results are critically dependent on these assumptions is an important avenue for future research—as is the question of whether the results generalize to a broader sample of languages.

Several other questions are left open by these findings. Importantly, given the centrality of communicative need to our analyses, do different cultures impose different communicative need distributions on the number line, and if so, do such cultural differences in need explain more cross-language variation in numeral systems than we have explained here? What sort of evolutionary process produces the diverse pattern in numeral systems? Future studies addressing these questions can help to place our present findings in their proper context. For now, however, our current work suggests that the functional drive for efficient communication may explain why we see particular numeral systems, and classes of numeral system, in the world’s languages.

## ACKNOWLEDGMENTS

We thank Charles Kemp for his role in developing the computational framework we use. We thank Stanislas Dehaene and Charles Kemp for comments on an earlier draft.

## FUNDING INFORMATION

This work was supported by NSF grant SBE-1041707 and DTRA grant HDTRA11710042 to TR and NSERC Discovery Grant RGPIN-2018-05872 to YX.

## AUTHOR CONTRIBUTIONS

YX: Conceptualization: Equal; Data curation: Lead; Formal analysis: Equal; Methodology: Equal; Writing–Original Draft: Equal; Writing–Review & Editing: Supporting. EL: Conceptualization: Supporting; Data curation: Supporting; Formal analysis: Equal; Methodology: Supporting; Writing–Original Draft: Supporting; Writing–Review & Editing: Equal. TR: Conceptualization: Equal; Data curation: Supporting; Formal analysis: Supporting; Methodology: Equal; Writing–Original Draft: Equal; Writing–Review & Editing: Equal.

## Notes

^1^ For example, different accounts of the Pirahã numeral system were presented by Gordon (2004) and by Frank et al. (2008), in large part because the two studies explored different numerical tasks. Specifically, Frank et al. (2008) examined both counting upward from a small number, and counting downward from a large number, whereas Gordon (2004) examined numeral use in a variety of contexts that did not include counting downward. We have chosen to use Gordon’s (2004) analysis in our cross-language study because downward counting has not been widely investigated across languages. We leave as an open question whether the principles we explore here will generalize to both forward and backward counting, across languages.^2^ The same loss function is used in rational speech act (RSA; e.g., Frank & Goodman, 2012) models in characterizing the utility of a speaker’s word choice.
