# Supplementary material for: Numeral Systems Across Languages Support Efficient Communication: From Approximate Numerosity to Recursion
Source: Open Mind (Camb). 2020 Aug 1;4:57–70. doi: 10.1162/opmi_a_00034 (PMC7685423; doi:10.1162/opmi_a_00034)
Supplement: Supplementary file 1 [file opmi-04-57-s001.pdf]

# Supplementary Materials
# Numeral systems across languages support efficient communication: From approximate numerosity to recursion

Yang Xu*[1], Emmy Liu[2], and Terry Regier[3]

[1]Department of Computer Science, Cognitive Science Program, University of Toronto
[2]Computer Science and Cognitive Science Programs, University of Toronto
[3]Department of Linguistics, Cognitive Science Program, University of California, Berkeley

## Contents

**1 Classes of numeral system** **1**

**2 Collection of numeral data** **2**

**3 Semantic primitives** **2**

**4 Numeral system grammars** **2**

**5 Listener distribution** **3**

**6 Modeling Mundurukú naming data** **4**

**7 Need probability** **6**

**8 Efficiency analyses with alternative Weber fractions** **7**

**9 Hypothetical numeral systems** **9**

**10 Complexities of canonical recursive systems** **10**

**11 Data and code availability** **12**

---

## 1 Classes of numeral system

Our definition of classes of numeral system largely follows that of Comrie (2013). Comrie draws a distinction between "restricted" numeral systems, which he defines as those that do "not effectively go above around 20", and other numeral systems, which cover a larger range, often through recursion. We took a language's numeral system to be *approximate* if the grammar or other description on which we relied for that language explicitly stated that the meanings of the numerals in the system are approximate or inexact. All such systems in our data were restricted in Comrie's sense. We took a language's numeral system to be *exact restricted* if the system covers a restricted range (again in Comrie's sense) but the description of the system did not explicitly state that the meanings were approximate or inexact; thus we assumed exactness unless there was evidence to the

*Correspondence: yangxu@cs.toronto.edu

contrary. Finally, we took a language's numeral system to be *recursive* if the numeral system was listed by Comrie as having a base which can be used to recursively produce numbers through a much higher range.

We classified each language based on the most fine-grained set of numeral terms available in the language, ignoring for now approximate terms in languages with an exact numeral system, e.g. "a few" in English. The classes of numeral system we consider do not perfectly partition the space of attested systems. For example, Comrie lists several extended body-part numeral systems, which use body parts beyond the 10 fingers to enumerate, and can reach well above 20, and there are some restricted languages that use recursion within a limited range. However, these broad classes do pick out major types of numeral system.

# 2 Collection of numeral data

For each of the 24 languages listed by Comrie (2013), we attempted to consult the reference work that Comrie lists for that language. There are several languages that are listed by Comrie (2013) but for which that chapter provides no reference. We located alternative references for each of these languages and those additional languages we analyzed, as listed below:

Chiquitano (Chan 2014: Chiquitano-South America)

English (Eastwood 1994: 245-247)

French (Chan 2014: French-Indo-European)

Fuyuge (Bradshaw 2007)

Krenak (Chan 2014: Krenak-South America)

Mandarin (Ross 2014: 28-29)

Pirahã (Gordon 2004: p496)

Spanish (Chan 2014: Spanish-Indo-European)

!Xóõ (Chan 2014: Khoisan-Africa)

We added to this set Pica et al.'s (2004) description of Mundurukú.

# 3 Semantic primitives

We explain here several of the semantic primitives out of which we construct grammars. The primitive concepts c=1, 2 or 3 are intended to capture the capacity for *subitizing*: the accurate estimation of small numbers up to about 3 (Revkin et al., 2008). $\tilde{x}$ is a Gaussian centered at position $\tilde{x}$ on a number line that scales in accord with the non-linguistic approximate number system, which obeys Weber's law; this primitive is intended to ground approximate numerals directly in that cognitive system. $\mathtt{s}(w,v)$ is a generalization of the standard successor function ($\mathtt{successor}(w) = \mathtt{m}(w)+1$); it defines an interval that begins at $\mathtt{m}(w)+1$ and continues for some exact length that is specified by the form $v$, i.e. the interval $[\mathtt{m}(w)+1, \mathtt{m}(w)+\mathtt{m}(v)]$. Although in attested systems the length $\mathtt{m}(v)$ of this line segment is generally 1, the more general interval case is used for hypothetical numeral systems against which we compare attested ones.[1] Finally, and again to support hypothetical systems, we also allow systems that are mirror-images of those definable in terms of these components: e.g. a standard one-two-many system would have numerals for 1, 2, and the range [3,100], whereas its mirror-image would instead have numerals for the range [1-98], 99, and 100.

# 4 Numeral system grammars

A typical reference work description for a given language's numeral system includes specification of the basic numerals (non-compositional forms, e.g. "one" to "twelve" in English), the bases of recursion if any (e.g. "ten" in English), and rules for composing higher numerals recursively out of the basic numerals and bases (e.g. "twenty-one" is defined as "two" times "ten" incremented by "one"). For each language, we translated such precise verbal descriptions of the numeral system into symbolic

[1]For example, in English, s("three","one") is [3+1,3+1] which picks out an interval containing just the integer 4, the successor of 3. In a hypothetical system based on English, one could instead have a numeral corresponding to s("three","two") = [3+1,3+2] which picks out the interval [4,5]; in this case the numeral would pick out a range containing two integers, not just a single integer.

form, cast in terms of the semantic primitive components in Table 2, resulting in full numeral grammars as in Table 5. For all languages, we restricted the grammatical specification to cardinal numerals that cover the range 1-100. We also assumed no languages contain any gap in that numerical interval. For example, for languages that do not have numerals up to 100, e.g. restricted systems, and contain a term that denotes "many" such as Pirahã, we defined the extension of such terms between the highest numeral preceding them and 100; for languages that do not have a "many" term listed in the reference work we consulted, we created a "many" category to fill the gap between the highest-order numeral available in that language and 100, hence assuming additional complexity for that category. For languages that contain multiple forms that denote the same number(s), we took the simplest form for that numeral in specifying a grammar. In addition, we constructed the grammar such that the meaning of every numeral has to be specified either by primitive concepts or in terms of meanings of numerals that are already defined.

The complexity of a grammar is defined as the total number of symbols needed to specify the entire grammar. This is the number of rules needed to specify each rule in the grammar, summed over all rules in the grammar. Consider the rule for the numerals 20...90 ("twenty"..."ninety") in the English grammar in Table 5:

$$\text{u`ty'} \stackrel{d}{=} \texttt{m(u)}\times\texttt{m(`ten')}$$

This rule has complexity 8, determined as follows: 1 symbol for the variable u, 1 for the form 'ty', 1 for the operator $\stackrel{d}{=}$, 1 for the operator $\texttt{m}(\cdot)$, 1 for the variable u, 1 for the operator $\times$, 1 for the operator $\texttt{m}(\cdot)$, and 1 for the form 'ten'. The complexity for each other rule in the grammar is determined analogously, and the complexity of the grammar as a whole is the sum of the complexities of its constituent rules.

# 5 Listener distribution

The listener distribution depends on the word $w$ uttered by the speaker, and thus depends on the primitives in terms of which that word is defined. We consider here listener distributions for words grounded in the subitizing number system, the approximate number system, and exact numerosity.

**Subitizing.** If the speaker has produced a word $w$ that is semantically grounded in the subitizing number system via a rule involving the primitive concepts 1, 2, or 3, we assume that the listener distribution takes the form:

$$L_w(i) \propto f(i|w) = \begin{cases} 1 & \text{(if number } i \text{ is named by } w) \\ 0 & \text{(otherwise)} \end{cases} \tag{1}$$

**Approximate number system.** If the speaker has produced a word $w$ that is semantically grounded in the approximate number system via a rule involving the primitive $\tilde{x}$, we assume that the listener distribution takes the form:

$$L_w(i) = p(i|w) \sim \mathcal{N}(i\,|\,\mu_w, \sigma_w^2) \tag{2}$$

This formulation follows from p. S5 of the supporting online materials from Pica et al. (2004), who present it as a formalization of the cognitive representation of numerosity in the non-linguistic approximate number system, which obeys Weber's law. $p(i|w)$ captures the listener's subjective degree of belief that the intended number is $i$, given that speaker has produced word $w$. The category corresponding to $w$ is represented as a normal distribution with mean $\mu_w = x$ and standard deviation $\sigma_w = v \times \mu_w$ following a scalar variability model, where $v$ is the empirically determined Weber fraction, which we take to be 0.31 in our analyses, following Piazza et al. (2013).[2]

**Exact numerosity.** In contrast, if the speaker has used an exact number term $w$ grounded in exact primitives such as $\texttt{s}(\cdot,\cdot)$, we assume that the listener distribution is uniform over numbers in the named interval:

$$L_w(i) = p(i|w) = \begin{cases} \frac{1}{|w|} & \text{(if } i \text{ is in category named by } w) \\ 0 & \text{(otherwise)} \end{cases} \tag{3}$$

where $|w|$ is the number of integers contained in the exact interval named by the number word $w$. In the case of most attested systems, an exact numeral such as "nine" will pick out just a single integer, so that $p(9|\text{"nine"}) = \frac{1}{1} = 1$. However the formula also generalizes to hypothetical exact numerals defined as longer exact intervals of the number line.

[2] We took this value at the suggestion of Stanislas Dehaene (personal communication). We also show below that our model is robust to variation in Weber fraction at alternative values of 0.25 and 0.15.

# 6 Modeling Mundurukú naming data

We obtained Mundurukú number naming data from Pica et al. (2004). Specifically, for numerosities 1 to 15, we noted the fraction of times each numerosity $i$ was named with a given Mundurukú word or locution $w$.[3] We modeled this fraction $p(w|i)$ using Bayes' rule: $p(w|i) \propto f(i|w)p(w)$, where the prior $p(w)$ is given by the relative frequency of word $w$ in the data, over all numerosities, and $f(i|w)$ is given by Equation 1 if $w$ is grounded in subitizing (which we assume for numeral categories that peak at 1, 2 or 3), or by Equation 2 if $w$ is grounded in the approximate numeral system (which we assume for all other Mundurukú categories). We fit this model to the Pica et al. (2004) data by finding placements of category means $\mu_w$ that minimize the mean-squared-error (MSE) between model and data. The model fit was very good (MSE = 0.002). The same model without subitizing yielded a three-fold increase in error (MSE = 0.006),[4] and a variant of this model that was instead based only on exact numeral representation (Equation 3) performed much more poorly (MSE = 0.03). We illustrate these findings in Figure 1 where we took Weber fraction to be 0.31. For the standard model, grounded in subitizing and approximate numerosity, we also assessed model performance under alternative values of the Weber fraction, specifically 0.25 (which yielded MSE = 0.0027) and 0.15 (MSE = 0.0096), illustrated in Figure 2. These findings suggest that the model of the approximate number system given by Equations 1 and 2 provide a reasonable basis for grounding approximate numeral systems.

[3] The data for a given numerosity $i$ did not always sum to 1.0 because some infrequent terms were not reported. In our model, we accommodated this fact by introducing an unnamed dummy category corresponding to these unreported terms.

[4] Because the subitizing model fit empirical Mundurukú data the best, we used subitizing for this and other approximate systems (except for Pirahã) in the efficiency analysis.

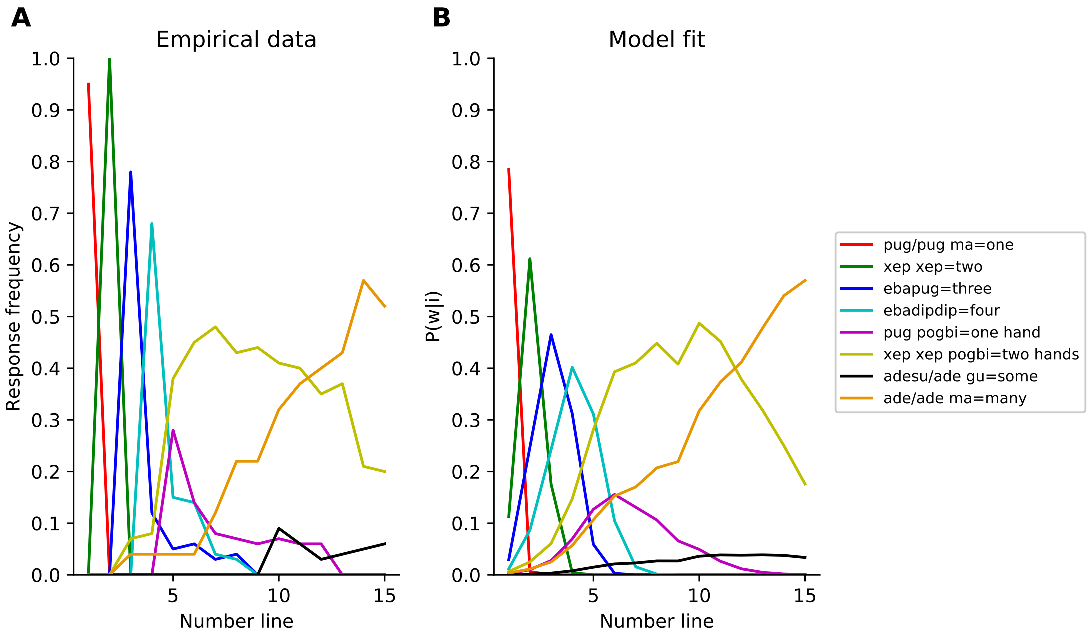


(a) Mundurukú model behaviour without subitizing.

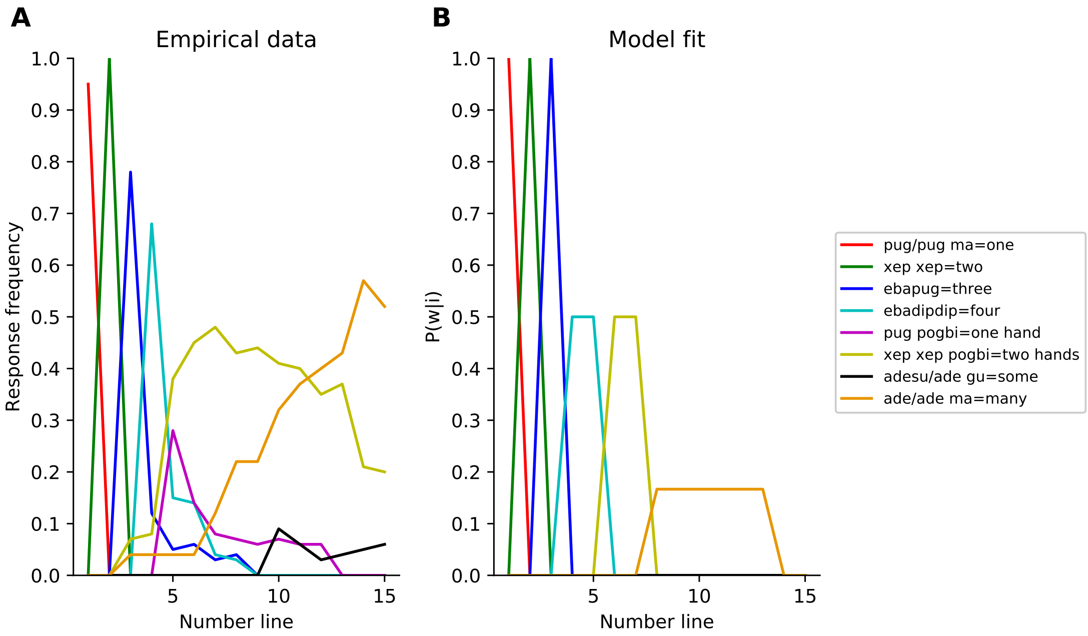


(b) Mundurukú model behaviour based on exact numeral representation.

Figure 1: Variants of the Mundurukú model (Weber fraction = 0.31) without subitizing and based on exact numeral representation (B), as compared to empirical data (A).

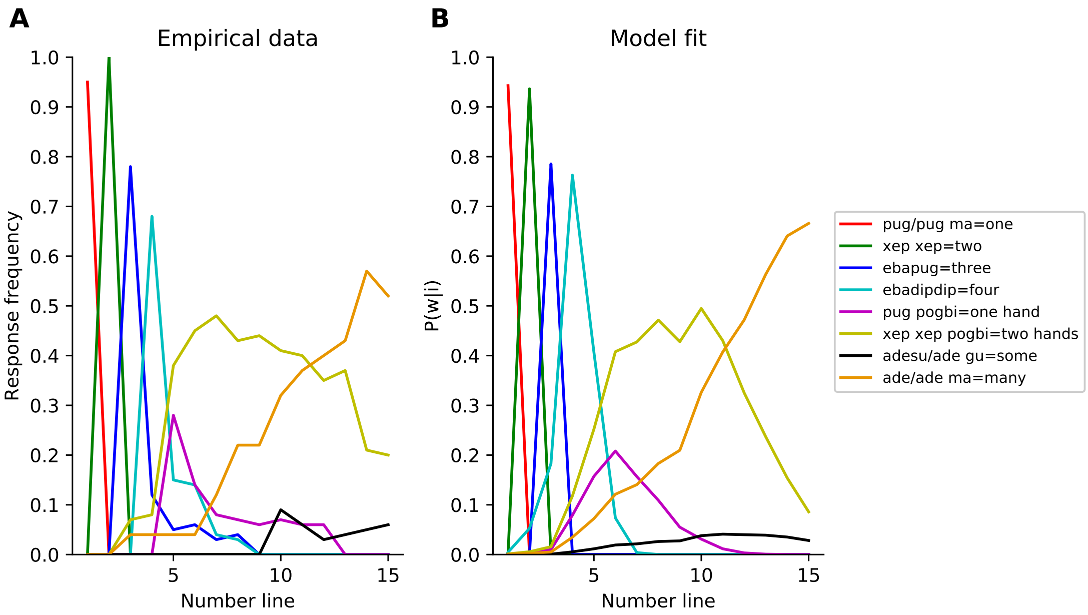


(a) Mundurukú model behaviour with subitizing (Weber fraction = 0.25).

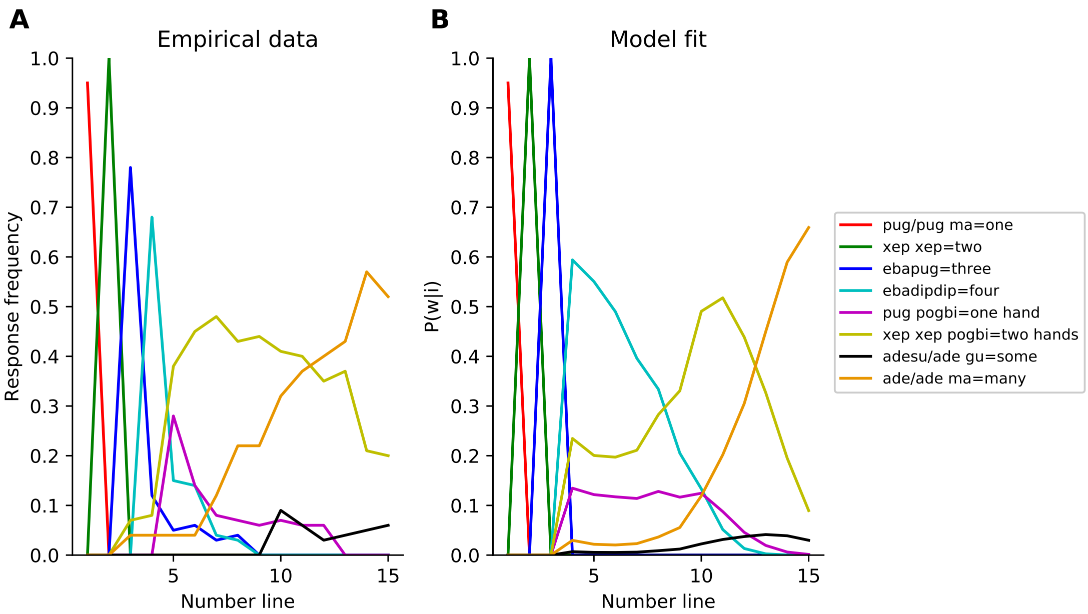


(b) Mundurukú model behaviour with subitizing (Weber fraction = 0.15).

Figure 2: Mundurukú model with subitizing at Weber fractions 0.25 (upper panel) and 0.15 (lower panel).

# 7 Need probability

We estimated need probabilities by the normalized frequencies of English numerals in the Google ngram corpus (Michel et al., 2011) for the year 2000, smoothed with a power-law distribution ($0.6182x^{-2.02}$; Pearson correlation with unsmoothed data = 0.97). Both the use of a power law, and the specific exponent we use here, are broadly consistent with earlier studies (Dehaene & Mehler, 1992; Piantadosi, 2016).[5] Figure 3 shows the raw and smoothed frequencies of English numerals in log-log scales. The Google ngram corpus is based on word and ngram frequencies in published books; numeral frequencies in spoken English

[5] In the main text, we also decreased the exponent to $-3$ to obtain a more left-skewed need distribution, and our results are qualitatively similar for English need probabilities without smoothing.

also decay with increasing target number $t$ (Leech et al., 2001).

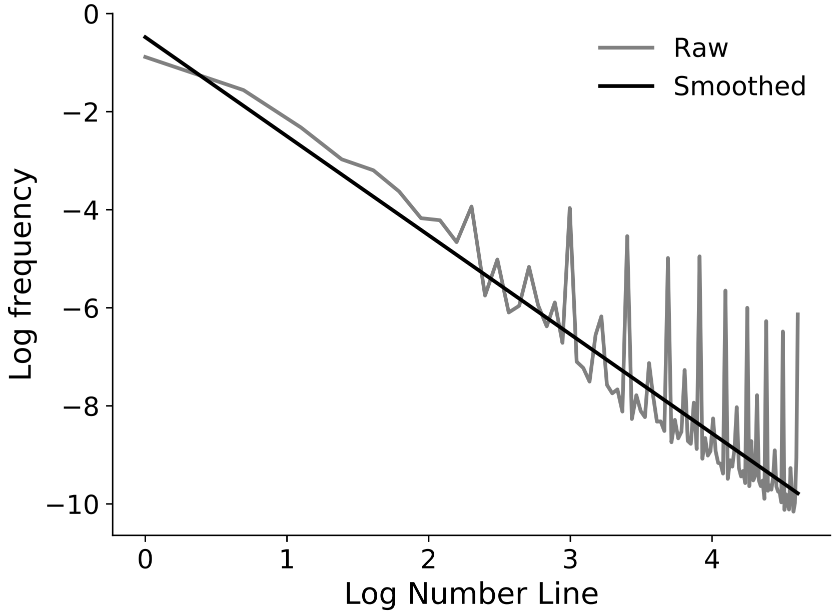


Figure 3: Raw and smoothed corpus frequencies of English numerals from 1 (“one”) to 100 (“hundred”).

# 8 Efficiency analyses with alternative Weber fractions

In the main text, we performed the efficiency analysis of numeral systems by assuming a Weber fraction of 0.31. Here we repeat this analysis under two differing Weber fractions: 0.25 and 0.15. Figures 4 and 5 show that our hypothesis holds robustly under variation of the Weber fraction.

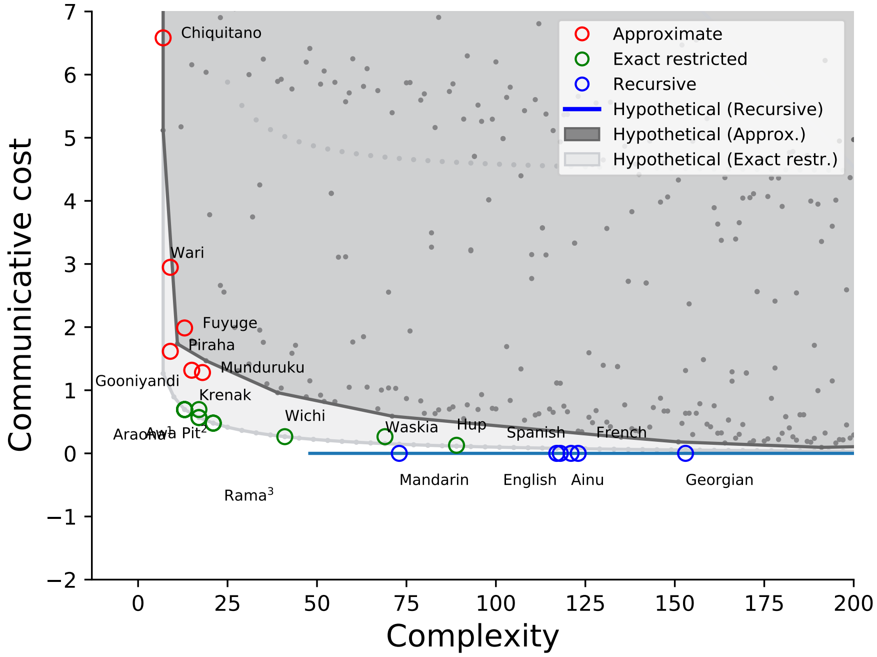


Figure 4: Efficiency analysis of numeral systems (Weber fraction = 0.25). Near-optimal tradeoff between communicative cost and complexity across attested numeral systems, compared with corresponding hypothetical approximate, exact restricted, and recursive systems.

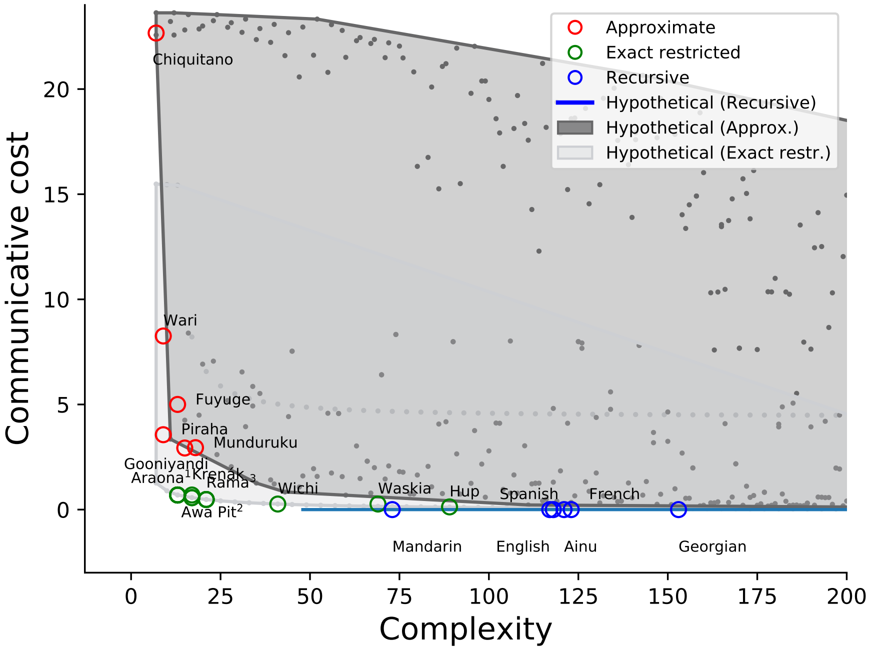


Figure 5: Efficiency analysis of numeral systems (Weber fraction = 0.15). Near-optimal tradeoff between communicative cost and complexity across attested numeral systems, compared with corresponding hypothetical approximate, exact restricted, and recursive systems.

# 9 Hypothetical numeral systems

We generated hypothetical numeral systems for each of the three major classes of system considered in this paper: approximate, exact restricted, and recursive.

**Hypothetical approximate systems.** To generate hypothetical approximate systems, we explored the space of possible approximate systems up to a maximum complexity of 200. Each such hypothetical approximate system is composed of some number $k$ of numeral categories, represented either as primitive concepts 1, 2, or 3 (subitizing) or as Gaussians. For each $k$, we initialized a hypothetical system by placing $k$ category centers (either means for Gaussians, or primitive concepts 1, 2, or 3) at random positions on the number line within the interval [1,100]. We then repeatedly adjusted the placement of each category center by shifting it either to the left or to the right on the number line by step size 1, if that shift lowered the communicative cost at that complexity, until no further local optimization was possible. We ran this greedy procedure 100 times, from 100 different initialization states, to alleviate the problem of locally optimal solutions.[6] We took all systems encountered during these optimization processes to be hypothetical approximate systems. The resulting grammar for each such hypothetical system was a list of Gaussians `g(·)` centered at these means, either combined with subitizing (e.g. Mundurukú) if numerals map to unique numbers up to 3, or without subitizing (e.g. Pirahã).

**Hypothetical exact restricted systems.** In the case of hypothetical exact restricted systems, we estimated the range of possible costs at each complexity—again up to complexity 200—by separately considering systems that should be expected to perform especially well, and those that should be expected to perform especially poorly. Because of the shape of the need probability distribution, we expect good performance (low cost) for systems that assign a separate single numeral to each integer on the

[6] To ensure our greedy procedure for exploring the space of hypothetical systems is valid, we also independently generated a more exhaustive set of systems in the range that accommodates the attested systems in our dataset. Specifically, we examined systems that have $k = 2$ through $k = 20$ numeral categories, and place these categories at the lower end of the number line in the interval [1,20]. We then enumerated all possible placements of $k$ means for a $k$-term system in the interval [1,20], producing $\binom{20}{k}$ systems for each $k$. This exhaustive procedure over a limited range yielded results similar to those from our greedy optimization procedure, which we extended over a wider range of complexities.

number line up to a numerical value k, and a terminal numeral that covers the remaining tail region up to 100. $k$ is varied from 2 to 99, yielding system of different complexities. Such systems place the only uninformative (and thus costly) large category in the least-weighted (high numerosity) part of the number line, and for this reason should perform well. We also considered mirror-images of these systems, that are expected to perform especially poorly, by analogous reasoning: these systems place numerals with large extensions at the beginning of the number line as opposed to at the tail.[7] The grammar for each such hypothetical system was a list of successor functions `s(·,·)` with varying interval lengths combined with subitizing up to 3, by analogy with the Kayardild grammar given in the main text. For each complexity, we assumed that the range of achievable costs by an exact restricted system was bounded by the high-performing and low-performing hypothetical system that we considered at that complexity.

**Hypothetical recursive systems.** Finally, we generated hypothetical recursive systems by considering the full space of canonical base-$n$ recursive numeral systems (Hurford, 1999) for $n$ = 2 to 100. We took a canonical base-$n$ system to be one in which there are distinct lexical items for the numerals 1 through $n$, and all numerals beyond that are constructed by generative rules according to recursive base-$n$ patterns such as $xn + y$ for some already-defined numerals $x, y$ (Comrie, 2013). In these systems, all numerals correspond to specific integers. The English grammar provided in the main text is not perfectly canonical because the teens are part of a separate subsystem from other high numerosities.

# 10 Complexities of canonical recursive systems

The canonical numeral system we chose for recursive systems is as follows: up to the base, each numeral $N$ is expressed as $n$ where $n$ is the value of $N$ if in the subitizing range, or $s(n-1)$ otherwise. After the base, the two rules $uB \stackrel{d}{=}$ `m('u') x m('B')` and $vw \stackrel{d}{=}$ `m('v') + m('w')` were used, where $B$ is the base of the numeral system, and $u$ and $v$ are previously defined numbers. For powers of the base, the rule $B^n \stackrel{d}{=}$ `p(m('B'), m('n'))` was used. It is implicit in this system that previously generated terms can be substituted into these rules, implying that higher numerical terms are built from lower-valued terms. Rules were generated from values 1-100. Canonical base 10 is provided as an example grammar in Table 1 (compare with grammar for English in the main text), along with example grammars for base 5 (Table 2) and base 3 (Table 3).

Figure 6 shows the complexities of canonical systems with different bases. We observed that the optimal system in this simulation has base 5. Although base-5 systems have been attested in the world's languages (Epps et al., 2012), they are less frequent than the dominant base-10 systems (Comrie, 2013). This mismatch may be due to the fact that there are factors outside complexity that drive the dominance of base-10 systems. Understanding the complexities of different recursive systems in the world's languages is a topic for future research.

[7] To empirically verify the region of the space we explored is valid, we also independently generated hypothetical systems exhaustively within the range of complexities that is attested across the language sample we have. We did so by exploring all possible partitions (from $k = 2$ through $k = 20$ numeral categories) at the interval [1,20] and comparing the resulting systems against the attested ones. This exhaustive procedure over a limited range yielded results identical to those from our procedure described above, which we extended over a wider range of complexities.

Table 1: Grammar for canonical base 10 system.

| Number | Rule | Complexity |
|---|---|---|
| 1 | ‘one’ $\stackrel{d}{=}$ `1` | 3 |
| 2 | ‘two’ $\stackrel{d}{=}$ `2` | 3 |
| 3 | ‘three’ $\stackrel{d}{=}$ `3` | 3 |
| 4 | ‘four’ $\stackrel{d}{=}$ s(‘three’) | 4 |
| 5 | ‘five’ $\stackrel{d}{=}$ s(‘four’) | 4 |
| 6 | ‘six’ $\stackrel{d}{=}$ s(‘five’) | 4 |
| 7 | ‘seven’ $\stackrel{d}{=}$ s(‘six’) | 4 |
| 8 | ‘eight’ $\stackrel{d}{=}$ s(‘seven’) | 4 |
| 9 | ‘nine’ $\stackrel{d}{=}$ s(‘eight’) | 4 |
| 10 | ‘ten’ $\stackrel{d}{=}$ s(‘nine’) | 4 |
| 20, 30,..., 90 | d-P $\stackrel{d}{=}$ m(d) $\times$ m(P) | 8 |
| 11-19, 21-29, ..., 91-99 | M-N $\stackrel{d}{=}$ m('M') + m('N') | 8 |
| 100 | ‘hundred’ $\stackrel{d}{=}$ p(m(‘ten’),m(‘two’)) | 7 |
| | P $\in$ { ’ten’, ’hundred’ } | 3 |
| | d $\in$ { ’one’, ‘two’,...,‘eight’,‘nine’ } | 10 |
| | | $\sum = 73$ |

Table 2: Grammar for canonical base 3 system.

| Number | Rule | Complexity |
|---|---|---|
| 1 | ‘one’ $\stackrel{d}{=}$ `1` | 3 |
| 2 | ‘two’ $\stackrel{d}{=}$ `2` | 3 |
| 3 | ‘three’ $\stackrel{d}{=}$ `3` | 3 |
| 6, 9,..., 99 | d-P $\stackrel{d}{=}$ m(d) $\times$ m(P) | 8 |
| 4-5, 7-8,..., 97-98, 100 | M-N $\stackrel{d}{=}$ m('M') + m('N') | 8 |
| 9 | ’9’ $\stackrel{d}{=}$ p(m(‘three’),m(’two’)) | 7 |
| 27 | ’27’ $\stackrel{d}{=}$ p(m(‘three’),m(’three’)) | 7 |
| 81 | ’81’ $\stackrel{d}{=}$ p(m(‘three’),m(’three-one’)) | 7 |
| | P $\in$ { ’three’, ’9’, ’27’, ’81’} | 4 |
| | d $\in$ { ’one’, ’two’ } | 3 |
| | | $\sum = 53$ |

Table 3: Grammar for canonical base 5 system.

| Number | Rule | Complexity |
|---|---|---|
| 1 | 'one' $\overset{d}{=}$ `1` | 3 |
| 2 | 'two' $\overset{d}{=}$ `2` | 3 |
| 3 | 'three' $\overset{d}{=}$ `3` | 3 |
| 4 | 'four' $\overset{d}{=}$ `s`('three') | 4 |
| 5 | 'five' $\overset{d}{=}$ `s`('four') | 4 |
| 10, 15,..., 95 | d-P $\overset{d}{=}$ `m`(d) × `m`(P) | 8 |
| 6-9, 11-14,..., 96-99 | M-N $\overset{d}{=}$ `m('M') + m('N')` | 8 |
| 25 | '25' $\overset{d}{=}$ `p(m(`'five'`),m(`'two'`))` | 7 |
| | P ∈ { 'five', '25'} | 3 |
| | d ∈ { 'one', 'two', 'three', 'four' } | 5 |
| | | $\sum = 48$ |

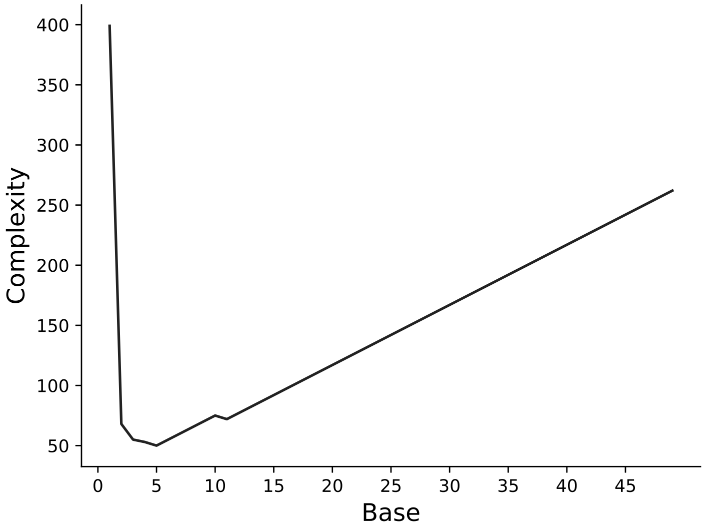


Figure 6: Complexity of canonical recursive numeral systems with different bases.

## 11 Data and code availability

Data and code for the analyses that we reported are available at `https://osf.io/jmrqw/?view_only=7fa3c3d085c743998cd8b1ebe92d74b4`.

# References

Bradshaw, R. L. (2007). *Fuyug grammar sketch.* SIL-PNG Academic Publications.

Chan, E. (2014). *Numeral systems of the world's languages. http://lingweb.eva.mpg.de/numeral/.* (Accessed: 2014-03-06)

Comrie, B. (2013). Numeral bases. In M. S. Dryer & M. Haspelmath (Eds.), *The world atlas of language structures online.* Leipzig: Max Planck Institute for Evolutionary Anthropology. (Available online at http://wals.info/chapter/131, Accessed on 2014-01-26)

Dehaene, S., & Mehler, J. (1992). Cross-linguistic regularities in the frequency of number words. *Cognition*, *43*(1), 1–29.

Eastwood, J. (1994). *Oxford guide to English grammar.* Oxford University Press.

Epps, P., Bowern, C., Hansen, C. A., Hill, J. H., & Zentz, J. (2012). On numeral complexity in hunter-gatherer languages. *Linguistic Typology*, *16*(1), 41–109.

Gordon, P. (2004). Numerical cognition without words: Evidence from Amazonia. *Science*, *306*, 496–499.

Hurford, J. (1999). Artificially growing a numeral system. In J. Gvozdanovic (Ed.), *Numeral types and changes worldwide.* Berlin: De Gruyter.

Leech, G., Rayson, P., & Wilson, A. (2001). *Word frequencies in written and spoken English based on the British National Corpus.* London: Longman.

Michel, J. B., Shen, Y. K., Aiden, A. P., Veres, A., Gray, M. K., The Google Books Team, ... Aiden, E. L. (2011). Quantitative analysis of culture using millions of digitized books. *Science*, *331*, 176–182.

Piantadosi, S. T. (2016). A rational analysis of the approximate number system. *Psychonomic Bulletin and Review*, 1–10.

Piazza, M., Pica, P., Izard, V., Spelke, E. S., & Dehaene, S. (2013). Education enhances the acuity of the nonverbal approximate number system. *Psychological Science*, *24*(6), 1037–1043.

Pica, P., Lemer, C., Izard, V., & Dehaene, S. (2004). Exact and approximate arithmetic in an Amazonian indigene group. *Science*, *306*, 499–503.

Revkin, S. K., Piazza, M., Izard, V., Cohen, L., & Dehaene, S. (2008). Does subitizing reflect numerical estimation? *Psychological Science*, *19*(6), 607–614.

Ross, C., & Ma, J. S. (2014). *Modern Mandarin Chinese grammar.* Routledge.
